# Diarylethenes Display In Vitro Anti-TB Activity and Are Efficient Hits Targeting the *Mycobacterium tuberculosis* HU Protein

**DOI:** 10.3390/molecules22081245

**Published:** 2017-07-25

**Authors:** María Angélica Suarez, Jhesua Valencia, Christian Camilo Cadena, Raktim Maiti, Chandreyee Datta, Gloria Puerto, José Hipólito Isaza, Homero San Juan, Valakunja Nagaraja, Juan David Guzman

**Affiliations:** 1 Departamento de Química y Biología, División de Ciencias Básicas, Universidad del Norte, Km 5 vía Puerto Colombia, Barranquilla 081007, Colombia; angelica.suarez91@gmail.com (M.A.S.); jhesua1025v@gmail.com (J.V.); 2 Departamento de Medicina, División de Ciencias de la Salud, Universidad del Norte, Km 5 vía Puerto Colombia, Barranquilla 081007, Colombia; christiancadenacruz@gmail.com (C.C.C.); hsanjuan@uninorte.edu.co (H.S.J.); 3 Department of Microbiology and Cell Biology, Indian Institute of Science, Bangalore 560012, India; raktimmaiti@gmail.com (R.M.); chandreyee@mcbl.iisc.ernet.in (C.D.); vraj@mcbl.iisc.ernet.in (V.N.); 4 Laboratorio de Micobacterias, Instituto Nacional de Salud, Calle 26 No. 51-20-Zona 6 CAN, Bogotá 111321, Colombia; gpuerto@ins.gov.co; 5 Grupo de Investigación en Productos Naturales y Alimentos (GIPNA), Departamento de Química, Universidad del Valle, Cali 760032, Colombia; jose.isaza@correounivalle.edu.co

**Keywords:** diarylethenes, stilbenes, tuberculosis, cytotoxicity, HU protein

## Abstract

Tuberculosis continues to be a great source of concern in global health because of the large reservoir of humans infected with the bacilli and the appearance of clinical isolates resistant to a wide array of anti-tuberculosis drugs. New drugs with novel mechanisms of action on new targets are urgently required to reduce global tuberculosis burden. *Mycobacterium tuberculosis* nucleoid associated protein (NAP) HU has been shown to be druggable and essential for the organism’s survival. In this study, four diarylethenes were synthesized using a one-pot decarboxylated Heck-coupling of coumaric acids with iodoanisoles. The prepared compounds **1**–**4** were tested for their in vitro growth inhibition of *M. tuberculosis* H37Rv using the spot culture growth inhibition assay, displaying minimum inhibitory concentrations between 9 and 22 µM. Their cytotoxicity against BHK-21 cell line showed half inhibition at concentrations between 98 and 729 µM. The most selective hit (SI = 81), demonstrated inhibition of *M. tuberculosis* HU protein involved in maintaining bacterial genome architecture.

## 1. Introduction

Tuberculosis (TB) is a bacterial disease caused by infection with members of the *Mycobacterium tuberculosis* complex, and is currently the leading cause of death from a single infectious agent in the world. Globally, 1.4 million people died from the disease and 10.4 million people were diagnosed with it in 2015 [1]. The main problem with TB disease is the large reservoir of infected individuals harbouring dormant bacilli, which are non-infectious and asymptomatic, but whom may develop active disease. It has been estimated that one third of global human population hide latent TB bacteria in a non-replicative stage called latent TB infection, and around 5–15% of this population will develop clinical signs of the disease during their lifetime [2]. If the host conditions are permissive, the bacteria will start to replicate, the host will develop active TB and the bacterium may spread to other hosts. The current TB chemotherapy is lengthy and complex, and some patients stop taking the drugs, mainly due to the fear of side effects, but also lack of access, toxicity, stigma, lack of trust in health care providers and other reasons [3]. If the bacterium is resistant to the first-line drugs, the treatment could even last for 2 years. Moreover a number of clinical isolates have been found to be resistant to almost all the anti-TB drugs [4]. There is no doubt that to attack these persistent and resistant bacterial forms, chemical drugs with novel mechanisms of action are urgently required.

Diarylethenes consist of two regioisomers, the 1,1-diarylethene and 1,2-diarylethene, the latter also known as stilbene. Stilbenes occur naturally in different botanical families such as the Vitaceae, Fabaceae, Pinaceae, among others. When bacterial, fungal or viral infection occurs, some plants quickly produce chemical defense molecules known as phytoalexins [5]. The most famous stilbene, resveratrol, is the phytoalexin of grapevine. A number of stilbenes have demonstrated anti-TB activity, for example the naturally occurring lakoochins [6] or the synthetic aza-stilbenes [7], which displayed growth inhibition in the micromolar range. The 1,1-diarylethenes have not been reported, to our knowledge, to display antimycobacterial properties. Although stilbenes have been found to inhibit the growth of the TB bacilli, there is little information about its mechanism of action and its target pathway or protein. A major nucleoid-associated *M. tuberculosis* HU protein (encoded by *hupB*, Rv2986c), which controls genome compaction and chromosomal architecture, has been found to be inhibited by stilbenes [8]. Taking cue from the above observation and in continuation of the study on the anti-TB effect of 2-hydroxycinnamic acid [9], three stilbenes and one 1,1-diarylethene were prepared from coumaric acids in a one-pot fashion, and tested for in vitro anti-TB activity and cytotoxicity against a mammalian cell line. The prepared diarylethenes were also screened for inhibition of the *M. tuberculosis* HU protein.

## 2. Results

### 2.1. Synthesis of Diarylethenes ***1***–***4***

**Scheme 1:**
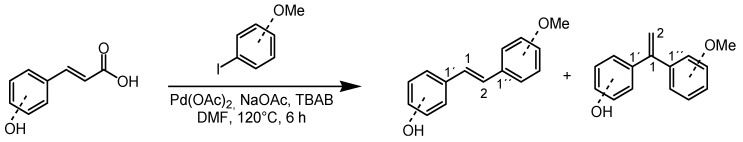
Decarboxylative one-pot Heck reaction of hydroxycinnamic acids with iodoanisoles under Jeffery conditions.

Three 1,2-diarylethenes **1**–**3** and one 1,1-diarylethene **4** were prepared using the one-pot decarboxylative coupling of coumaric acids with iodoanisoles (Scheme 1) under ligandless Jeffery conditions [Pd(OAc)_2_, tetrabutylammonium bromide and inorganic base] [10]. 2′-Hydroxy-4′′-methoxy-1,2-diarylethene (**1**) was obtained from the corresponding coumaric acid in 43% yield on a 1.5 mmol scale after purification by silica gel column chromatography. The yields for the synthesis of the diarylethenes **2**, **3** and **4** were respectively 32%, 44% and 44%. The diarylethenes **1**–**4** are not novel and their NMR and MS spectra are in agreement with previous reports in the literature [11,12].

### 2.2. Antituberculosis Activity and Cytotoxicity of Diarylethenes ***1***–***4***

All the four diarylethenes **1**–**4** were tested for *M. tuberculosis* H37Rv growth inhibition using the spot culture growth inhibition assay [9,13]. The minimum inhibitory concentration (MIC) values of the diarylethenes having the 2′-hydroxy substitution **1**, **2** and **4** were 9.0 µM, while the diarylethene **3** with a 4′-hydroxy substitution was less active with MIC value of 22 µM (Table 1). Interestingly, a similar effect was also observed for the coumaric acids, being the 2-hydroxy substituted the most active against *M. tuberculosis* [9]. The cytotoxicity against the normal (non-cancer) baby hamster kidney cells (BHK21) using 3-(4,5-dimethylthiazol-2-yl)-2,5-diphenyltetrazolium bromide (MTT) viability assay [14], showed that the 2′-hydroxydiarylethenes were the less toxic with half-growth inhibitory concentration (GIC_50_) values ranging between 499 and 729 µM (Table 1). The diarylethene with a 4′-hydroxy substitution (**3**) was much more toxic with a GIC_50_ value of 98 µM. The selectivity index (SI) is calculated as the ratio between GIC_50_ and MIC values. A compound showing an SI value higher than 10 is considered to have a favourable toxicity profile [15], and may progress to an infection assay to confirm its activity.

### 2.3. Inhibition of Mtb-HU Protein and Docking

**Figure 1:**
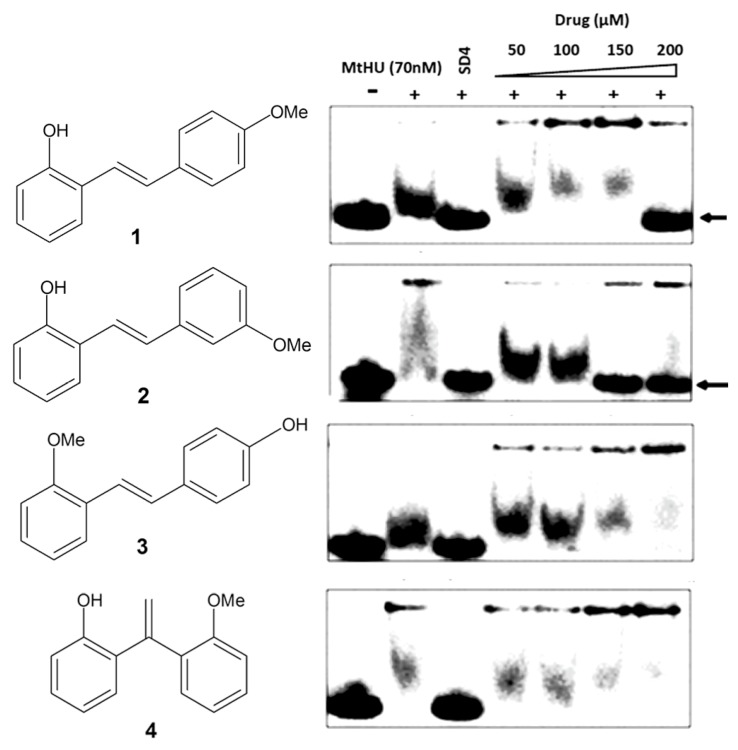
Chemical structures of the prepared diarylethenes **1**–**4** and their inhibition of *Mycobacterium tuberculosis* HU binding to DNA. Lane − is for pure protein and lane + is for the mixture of HU and DNA showing complex formation. SD4 is an experimental HU inhibitor [8] used a positive control at 5 µM concentration.

The diarylethenes **1**–**4** were evaluated for their inhibition of DNA-HU binding by electrophoretic mobility shift assays. The HU protein binds to DNA substrates and forms stable DNA-HU complexes. In the presence of inhibitors, the DNA-HU complexes do not form. Both 2′-hydroxy-1,2-diarylethenes **1** and **2**, showed inhibition of Mtb-HU binding to DNA (Figure 1), although **2** was slightly more active than **1**. The Mtb-HU inhibitory concentration (IC_90_) for compounds **1** and **2** was determined to be 200 and 150 µM respectively. The other diarylethenes **3** and **4**, were not inhibitory to DNA-HU binding up to 200 µM.

**Figure 2:**
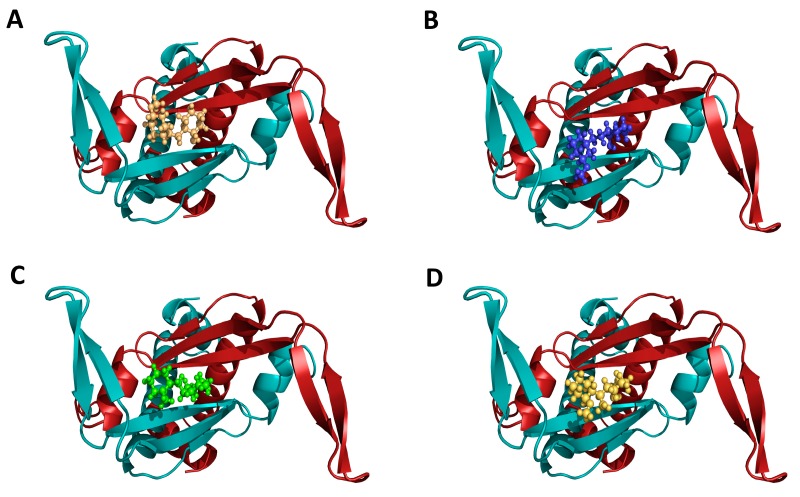
Top view of the docked poses of the diarylethenes **1** (**A**), **2** (**B**), **3** (**C**), **4** (**D**) with the DNA binding saddle of *Mycobacterium tuberculosis* HU protein.

All the diarylethenes were docked (Figure 2) on the published *M. tuberculosis* HU crystallographic structure (PDB: 4PT4) [8]. The docking scores calculated using Glide extra-precision, are given in Table 1. According to the docking study, **2** binds to HU most efficiently (Glide score = −3.292), followed by **1** (Glide score = −3.266). The other two diarylethenes showed lower docking scores indicating lower affinity for HU.

## 3. Discussion

When 2-hydroxycinnamic acid was coupled with 4-iodoanisole or 3-iodoanisole, or when 4-hydroxycinnamic acid was reacted with 2-iodoanisole, the major products were the corresponding 1,2-diarylethenes, whereas when the coupling was performed with 2-hydroxycinnamic acid and 2-iodoanisole, the major product was the 1,1-diarylethene. This lack of regioselectivity is explained by the Jeffery conditions with unliganded Pd catalyst, which at high temperature converts quickly to the insoluble palladium black, yielding undesired products or no conversion [16]. The aim of this study was to prepare diarylethenes in sufficient amount to be evaluated for anti-TB activity and cytotoxicity, and thus the optimization of the conditions for the one-pot reaction were not performed. In addition, the synthetic route failed to yield Heck products when using iodophenols, but it worked relatively well with iodoanisoles. We are currently exploring the use of an *N*-heterocycle Pd ligand to yield specific 1,1- or 1,2-diarylethenes and the results will be reported in a future communication.

Among the prepared diarylethenes **1**–**4**, the most selective molecule identified was 2′-hydroxy-3′′-methoxy-1,2-diarylethene (**2**) with a SI of 81. This is the highest SI value achieved for a simple cinnamic acid derivative in TB drug discovery [17]. In addition, this is the first time that a 1,1-diarylethene (compound **4**) is found to display potent anti-TB activity, in a similar order of magnitude as 1,2-diarylethenes (stilbenes), which are well-known antimicrobial molecules. Diarylethenes bearing a 2′-hydroxy substitution **1** and **4**, showed lower SI values (58 and 55 respectively). The 4′-hydroxydiarylethene **3** was less selective towards mycobacteria with an SI value of 4.4. Interestingly an aza-stilbene with the same substitution on the aromatic rings as **1**, showed an MIC value against the H37Rv strain of 6.9 µM and SI of 113 with reference to Vero cells [18]. This result is in agreement with our study, and it suggests that the chemical space of 2′-hydroxydiarylethenes is sufficiently selective for continuing efforts to optimize this hit against TB.

It was interesting to note that the stilbenes showing HU inhibition (**1** and **2**), displayed lower MIC values and the highest selectivity among the screened compounds, but also the best docking scores. In the earlier study [8], the stilbenes SD1 and SD4 inhibited the HU protein with respective IC_90_ values of 1.7 and 20 µM, showing to be more potent than stilbenes **1** and **2**. However the compounds SD1 and SD4 were larger than **1** and **2**, and their calculated ligand efficiency (LE) [19] was respectively 2.5 and 1.9, while the LE for **1** and **2** was calculated to be 5.3 and 5.5 respectively at 25 °C. Therefore, these stilbene derivatives display higher affinity per atom, and have the potential to be further developed as efficient inhibitors of HU of *M. tuberculosis*. Future work will enhance the potency and selectivity of both 1,1- and 1,2-diarylethenes and verify the proposed target by assessing with detail the mechanism of action of this class of anti-TB molecules.

## 4. Materials and Methods

### 4.1. Reagents, Strains and Equipment

The reagents and solvents were purchased respectively from Sigma (St. Louis, MO, USA) and Merck (Darmstadt, Germany). *Mycobacterium tuberculosis* H37Rv (ATCC 27294) is the reference strain at the Instituto Nacional de Salud (Bogotá, Colombia). Baby hamster kidney (BHK-21) cells (EH1011) were purchased from Kerafast (Boston, MA, USA). The mass spectrometry analysis was performed on a ThermoFisher Trace 1310 gas chromatograph coupled to a Thermo ISQ mass detector (ThermoFisher, Waltham, MA, USA) operated in 1:20 split mode, in a 100% Rxi-1 polydimethylsiloxane capillary column (30 m × 0.25 mm × 0.50 µm, Restek, Bellefonte, PA, USA) using helium as carrier gas at 1.0 mL/min. Mass spectra were obtained by electron ionization at 70 eV in a quadrupole mass detector with a mass range between 40 and 500 *m*/*z* in the full scan mode. Nuclear magnetic resonance spectra were acquired on an Ultrashield 400 spectrometer (Bruker, Billerica, MA, USA) on an Advance II workstation using deuterated acetone as solvent.

### 4.2. Synthesis of Diarylethenes

The appropriate hydroxycinnamic acid (1.05 equivalents) and the iodomethoxyaryl compound (1.0 equivalent) were dissolved in dimethylformamide (DMF, 5–10 mL), and mixed with the phase-transfer reagent tetrabutylammonium bromide (TBAB, 1.0 equivalent), the base sodium acetate (2.0 equivalents), and the palladium acetate catalyst (0.03 equivalents). The mixture was stirred at 120 °C for 6 h, then allowed to cool to room temperature and around 10 mL of 0.1 M HCl were added. The obtained mixture was extracted with ethyl acetate and then dried over anhydrous magnesium sulphate, filtered and the solvent removed under reduced pressure to yield a crude product. Silica gel column chromatography was used to obtain the pure compounds using dichloromethane as mobile phase.

*(E)-2*′*-Hydroxy-4′′-methoxy-1,2-diarylethene* (**1**): ^1^H-NMR: 7.59 (1H, dd, *J* = 7.6, 1.6 H(6′)), 7.52 (2H, m, H(2′′,6′′)), 7.40 (1H, d, *J =* 16.5 H(1)), 7.19 (1H, d, *J =* 16.5 H(2′)), 7.08 (1H, ddd, *J =* 8.6, 7.4, 1.7 H(4′)), 6.95 (2H, m, H(3′′,5′′)), 6.92 (1H, dt, *J* = 8.5, 1.1 H(5′)), 6.85 (1H, td, *J* = 7.5, 1.2 H(3′)), 3.83 (3H, s, OCH_3_).^13^C-NMR: 160.3 (C(4′′)), 155.6 (C(2′)), 131.8 (C(1′′)), 129.0 (C(4′)), 128.9 (C(2)), 128.5 (C(2′′,6′′)), 127.2 (C(6′)), 125.7 (C(1′)), 122.4 (C(1)), 120.8 (C(3′)), 116.6 (C(5′)), 115.0 (C(3′′,5′′)), 55.7 (C(OCH_3_)). MS/EI (*m*/*z*): 226 (M^+●^, 100), 165 (34), 211 (30), 118 (12), 183 (11), 77 (10).

*(E)-2′-Hydroxy-3′′-methoxy-1,2-diarylethene* (**2**): ^1^H-NMR: 7.62 (1H, dd, *J* = 7.61, 1.37 H(6′)), 7.57 (1H, d, *J =* 16.59 H(1)), 7.27 (1H, m, (5′′)), 7.23 (1H, d, *J =* 16.7 H(2)), 7.17 (2H, m, H(2′′, 6′′)), 7.11 (1H, m, H(4′)), 6.96 (1H, m, *J* = 7.41 H(3′)), 6.89 (1H, m, H(5′)), 6.84 (1H, m, H(4′′)) 3.83 (3H, s, OCH_3_). ^13^C-NMR: 161.2 (C(3′′)), 155.9 (C(2′)), 130.5 (C(5′′)), 129.6 (C(4′)), 129.3 (C(2)), 129.2 (C(1′′)), 127.6 (C(6′)), 125.3 (C(1′)), 125.0 (C(1)), 120.8 (C(5′)), 119.8 (C(6′′)), 116.7 (C(3′)), 114.0 (C(4′′)), 112.5 (C(2′′)), 55.6 (C(OCH_3_)). MS/EI (*m*/*z*): 226 (M^+●^, 100), 165 (74), 195 (50), 225 (47), 211 (45), 209 (44), 227 (40).

*(E)-4′-Hydroxy-2′′-methoxy-1,2-diarylethene* (**3**): ^1^H-NMR: 7.61 (1H, d, *J* = 7.61 H(3′′)), 7.43 (2H, d, *J* = 8.5 H(2′,6′)), 7.33 (1H, d, *J* = 16.39 H(1)), 7.21 (1H, m, *J* = 8.39 H(4′′)), 7.14 (1H, d, *J* = 16.59 H(2)), 6.99 (1H, m, *J* = 8.2 H(5′′)), 6.94 (1H, m, *J* = 7.6 H(6′′)), 6.86 (2H, d, *J* = 8.9 H(6′,2′)), 3.88 (3H, s, OCH_3_).^13^C-NMR: 158.1 (C(2′′)), 157.8 (C(4′)), 130.7 (C(1′)), 129.7 (C(4′′)), 129.1 (C(2)), 128.7 (C(3′,5′)), 127.6 (C(1′′)), 126.8 (C(3′′)), 121.6 (C(6′′)), 121.3 (C(1)), 116.5 (C(2′,6′)), 112.0 (C(5′′)), 56.0 (C(OCH_3_)). MS/EI (*m*/*z*): 226 (M^+●^, 100), 119 (45), 120 (43), 165 (40), 183 (22), 227 (16).

*(E)-2′-Hydroxy-2′′-methoxy-1,1-diarylethene* (**4**)*:* ^1^H-NMR: 7.64 (1H, dd, *J =* 7.68, 1.37 H(3′′)), 7.57 (1H, dd, *J* = 7.41, 1.56 H(6′)), 7.52 (2H, s, H(2)), 7.24 (1H, dt, *J* = 7.61, 1.76 H(4′′)), 7.10 (1H, dt, *J =* 7.41, 1.56 H(4′)), 7.00 (1H, m, *J* H(5′′)), 6.97 (1H, m, H(6′′)), 6.92 (1H, m, H(3′)), 6.86 (1H, m, H(5′)), 3.88 (3H, s, OCH_3_).^13^C-NMR: 157.9 (C(2′′)), 155.7 (C(2′)), 129.4 (C(4′′)), 129.3 (C(4′)), 127.9 (C(1′′)), 126.9 (C(3′′)), 126.0 (C(1′)), 124.8 (C(1)) 123.8 (C(2)), 121.6 (C(6′′)), 120.8 (C(5′)), 116.6 (C(3′)), 112.1 (C(5′′)), 56.0 (C(OCH_3_)). MS/EI (*m*/*z*): 226 (M^+●^, 100), 119 (49), 91 (46), 165 (37), 120 (27), 183 (12).

### 4.3. Antituberculosis Activity

The anti-TB activity was evaluated at the accredited biosafety level 3 laboratory of the “Instituto Nacional de Salud (Bogotá)”, using the spot culture growth inhibition assay [9,13] in 24 well plates. Briefly, a stock solution of 200 mg/mL in DMSO of each compound was prepared, and then different volumes of the stock were dispensed into the sterile 24 well plates so that a final concentration between 2 and 200 mg/L was achieved with the final addition of 2 mL Middlebrook 7H10 (HiMedia, Mumbai, India) appropriately supplemented with glycerol and oleic acid, albumin, dextrose, catalase (OADC, BD, Franklin Lakes, NJ, USA). An inoculum containing a cell density of 10^6^ CFU/mL was prepared in sterile water from a Löwenstein-Jensen slant of *M. tuberculosis* H37Rv grown for 3–4 weeks. A volume of 2.0 µL of the inoculum was carefully dispensed into the middle of each well and the plate was incubated at 37 °C for 2–3 weeks. Isoniazid was included as a positive control. Plates were carefully observed and MICs were recorded as the minimum concentration of compound which completely inhibited bacterial growth by visual inspection.

### 4.4. Cytotoxicity

The baby hamster kidney (BHK-21) cells were grown in 4 mM l-glutamine DMEM high glucose media (Glutamax, ThermoFisher, Waltham, MA, USA) supplemented with 10% fetal bovine serum and 1% penicillin and streptomycin. The cells were cultivated in 15 mL of medium dispensed in 75 cm^2^ cell culture flask at 37 °C in a humidified incubator with 5% CO_2_, for 3–4 days. The cells were treated with 1.5 mL of filter-sterilized 0.25% trypsin-EDTA for 2 min and then neutralized with 10 mL of fresh media. The cells were resuspended and centrifuged at 300 *g* for 5 min, and then counted with trypan blue using a heamocytometer. The 96-well plate was then seeded with 10^4^ cells in 100 µL of media per well, and incubated overnight. The compounds were dissolved in sterile DMSO at a stock concentration of 200 g/L. In a separate 96-well plate, the compounds were diluted by transferring 2 µL of the stock in 220 µL of fresh medium to the first well and then serially diluting with 110 µL in each well. A volume of 100 µL of each dilution prepared in this way was dispensed into the 100 µL of the overnight culture, so that the final concentrations tested were from 909 to 0.44 mg/L. Mitmab was used as a positive control. The plates were incubated for 24 h and then 20 µL of a 0.22 µm filter sterilized MTT solution (2.5 g/L in sterile water) were added to each well. Following overnight incubation, the media was removed, and 50 µL of DMSO were added to each well. The plates were incubated for 30 min, and then absorbance was measured at 550 nm using a Synergy 2 microplate reader (Biotek, Winooski, VT, USA). The experiment was performed by duplicate.

### 4.5. Mtb-HU Inhibition

A 30-bp single stranded oligonucleotide was labelled at its 5′ end with [γ-32P] ATP using the T4 polynucleotide kinase. Double-stranded DNA substrate was prepared by annealing the unlabelled complementary oligonucleotide to the labelled one. The *M. tuberculosis* HU at 70 nM was incubated with the double stranded labelled DNA substrate on ice for 15 min. The compounds were dissolved in DMSO. The compounds **1**–**4** were added in increasing concentrations and further incubated for 5 min. SD4 was used at 5 µM as a positive control. All reactions were analysed in 6% (*w*/*v*) non-denaturing polyacrylamide gels (29.4:0.6 acrylamide:bisacrylamide) in TBE buffer [45 mM Tris−borate (pH 8.3) and 1 mM EDTA] and visualised using a phosphorimager (Fujifilm, Tokyo, Japan).

### 4.6. In-silico Docking with Mtb-HU

The initial topologies of the compounds were derived by using the Gaussian 09 package [20]. These lowest energy states were derived by DFT method [21] with 6-311G Basis set and B3LYP functional [22]. These 3D models were further used for docking study using Glide [23] with *M. tuberculosis* HU protein (PDB: 4PT4). The Maestro suite (Schrodinger LLC, New York, NY, USA) was used for pre-processing of the protein followed by energy minimization with OPLS force field. Multiple conformers of the ligands were generated using LigPrep and docked against *M. tuberculosis* HU using Glide with extra precision docking [24].

## 5. Conclusions

The stilbenes (*E*)-2′-hydroxy-4′′-methoxy-1,2-diarylethene (**1**) and (*E*)-2′-hydroxy-3′′-methoxy-1,2-diarylethene (**2**) showed to inhibit the growth of virulent *M. tuberculosis* H37Rv with an MIC value of 9.0 µM. Their SI values were respectively 58 and 81 in relation to normal baby hamster kidney cells (BHK21). In addition, this is the first time that a 1,1-diarylethene **4** was found to significantly inhibit the growth of *M. tuberculosis* (MIC = 9.0 µM) in a selective fashion (SI = 55). Among the synthetised diarylethenes, **1** and **2** displayed inhibition of *M. tuberculosis* HU-DNA binding, with respective IC_90_ values of 200 and 150 µM. Both 1,2-diarylethenes **1** and **2** are efficient inhibitors of HU-DNA complex formation, and they are considered favourable scaffolds for further development as anti-TB drugs.

## Figures and Tables

**Table 1:** Antituberculosis activity against *Mycobacterium tuberculosis* H37Rv, cytotoxicity of BHK21 mammalian cell line and Mtb-HU protein inhibition by the diarylethenes **1**–**4**.

Compound	MIC H37Rv (µM)	GIC_50_ BHK21 (µM)	Selectivity Index (SI)	IC_90_ Mtb-HU (µM)	Glide Docking Score
**1**	9.0	522	58	200	−3.26
**2**	9.0	729	81	150	−3.29
**3**	22	98	4.4	>200	−2.86
**4**	9.0	499	55	>200	−2.96
Isoniazid	3.6	nd	nd	nd	nd
Mitmab	nd	8.8	nd	nd	nd

nd stands for not determined.
